# A Lactate Kinetics Method for Assessing the Maximal Lactate Steady State Workload

**DOI:** 10.3389/fphys.2018.00310

**Published:** 2018-03-29

**Authors:** Gernot O. Hering, Ewald M. Hennig, Hartmut J. Riehle, Jens Stepan

**Affiliations:** ^1^Department of Sport and Health Science, University of Konstanz, Konstanz, Germany; ^2^Institute of Health and Biomedical Innovation, Queensland University of Technology, Brisbane, QLD, Australia

**Keywords:** lactate threshold, maximal lactate steady state workload, lactate kinetics, performance testing, exercise physiology

## Abstract

During a continuously increasing exercise workload (WL) a point will be reached at which arterial lactate accumulates rapidly. This so-called lactate threshold (LT) is associated with the maximal lactate steady state workload (MLSS_W_), the highest WL, at which arterial lactate concentration [LA] does not change. However, the physiological range in which the LT and the MLSS_W_ occur has not been demonstrated directly. We used minor WL variations in the MLSS_W_ range to assess arterial lactate kinetics in 278 treadmill and 148 bicycle ergometer exercise tests. At a certain workload, minimal further increment of running speed (0.1–0.15 m/s) or cycling power (7–10 W) caused a steep elevation of [LA] (0.9 ± 0.43 mM, maximum increase 2.4 mM), indicating LT achievement. This sharp [LA] increase was more pronounced when higher WL increments were used (0.1 vs. 0.30 m/s, *P* = 0.02; 0.15 vs. 0.30 m/s, *P* < 0.001; 7 vs. 15 W, *P* = 0.002; 10 vs. 15 W, *P* = 0.001). A subsequent workload reduction (0.1 m/s/7 W) stopped the [LA] increase indicating MLSS_W_ realization. LT based determination of running speed (MLSS_W_) was highly reproducible on a day-to-day basis (*r* = 0.996, *P* < 0.001), valid in a 10 km constant velocity setting (*r* = 0.981, *P* < 0.001) and a half marathon race (*r* = 0.969, *P* < 0.001). These results demonstrate a fine-tuned regulation of exercise-related lactate metabolism, which can be reliably captured by assessing lactate kinetics at the MLSS_W_.

## Introduction

Lactate concentration in the blood of healthy humans usually follows workload (WL) demands (Brooks, 1985a). This relationship significantly changes at the lactate threshold (LT), when an accelerated blood lactate accumulation in response to a minor WL increment occurs (Brooks, 1985a; Messonnier et al., 2013). The maximal lactate steady state workload (MLSS_W_) should be below the LT as the highest WL at which the rate of entry of lactate into the systemic circulation is equivalent to the rate of disappearance from the bloodstream (Messonnier et al., 2013). Yet for several decades, both parameters have been extensively used as biomarkers for endurance exercise capability and for the control of endurance training paradigms (Billat et al., 2003, 2004; Faude et al., 2009). Although previous research indicates a fine-tuned regulation of LT and MLSS_W_ appearance (Messonnier et al., 2013), the precise range in which they occur has not been demonstrated directly.

Since the first notion of a strong association between blood lactate levels during exercise and endurance capacity, the understanding and interpretation of its metabolism has been subject to dramatic change, which remains ongoing (Brooks and Gladden, 2003; Gladden, 2004; Brooks, 2014b). In the 1920s, Hill and Meyerhof developed the “O_2_ depth” theory (Hill and Lupton, 1923; Meyerhof, 1930; Brooks and Gladden, 2003; Brooks, 2014a; Nobelprize.Org, 2017) and provided an O_2_ deficiency explanation for lactate accumulation. Using the respiratory gas exchange ratio, this theory was taken up by Wasserman and co-workers in the mid-1960s (Wasserman and McIlroy, 1964; Wasserman et al., 1973), who also introduced the term “anaerobic threshold” (AT). In contrast, Stainsby and Welch already demonstrated 1966 in dog muscles that lactate production can occur independently from O_2_ delivery (Stainsby and Welch, 1966).

However, the model of lactate accumulation as a consequence of an O_2_ deficit remained popular in performance testing, until a growing body of data, reviewed in 1985 by Brooks (1985a), raised doubts about this simple explanation and the concept of the AT. An alternative theory for lactate accumulation and subsequent fatigue is based on research indicating that the post-steady state exponential increase of the lactate concentration is a consequence of a diminished lactate removal from the bloodstream (Donovan and Brooks, 1983; Stanley et al., 1985). Supporting evidence came from tracer experiments, demonstrating that the abrupt increase of arterial lactate concentration [LA] after the equilibrium between lactate appearance (La) and lactate disappearance (Ld) at the AT, is the result of an increased La/Ld co-efficient (Donovan and Brooks, 1983; Brooks, 1985a; Stanley et al., 1985). However, at the same time a well-recognized study by Heck et al. (1985) justified the so called “4 mM”—or “aerob-anaerobic threshold” first introduced by Mader et al. (1976), as a consequence of the inability to oxidatively covering energy needs following the initial production of lactate (Heck et al., 1985).

Like the research group around Brooks (Donovan and Brooks, 1983; Brooks, 1985b; Stanley et al., 1985), Heck et al (Heck et al., 1985) used the term “steady state” of lactate, and proposed the concept of the “Maximal Lactate Steady State” (Billat et al., 2003; Faude et al., 2009), to assess the highest exercise specific endurance workload, by determining the highest constant arterial lactate level (Heck et al., 1985).

Although the appropriate use of lactate measurements and data interpretation is still a matter of debate, the high correlation between the onset of plasma or blood lactate accumulation (OPLA, OBLA) and endurance performance already demonstrated in 1979 by Farrell et al. (1979) and 1981 by Sjödin and Jacobs (1981), supported the idea to use lactate biochemistry as a diagnostic tool. Since then, a number of lactate tests have been developed (Billat et al., 2003; Faude et al., 2009), but for economic solutions, most of them try to measure the MLSS_W_ with graded WL increments before accelerated blood lactate accumulation occurs (Billat et al., 2003; Faude et al., 2009). Moreover, the widely used WL increments of 0.3–0.5 m/s in running or 30–50 W in cycling respectively (Svedahl and MacIntosh, 2003), may constrain the detection of small metabolic events. The maximum variation of running velocity during the current official world marathon record (0.07 m/s) (Kimetto, 2014) and the fastest ever recorded marathon time (0.07 m/s; final 2.2 km 0.14 m/s) (Tucker, 2017) emphasizes the potential fine-tuned regulation of lactate metabolism (Messonnier et al., 2013). This suggests that elite athletes perform close to their MLSS_W_, and that minor WL deviations may result in a rapid decline of running speed or power output.

Usually, several tests on different days are needed for a precise determination of the MLSS_W_ (Heck et al., 1985; Faude et al., 2009). Here, we report a reliable and valid test method for running and cycling, which employs lactate kinetics to detect the LT and to subsequently determine the MLSS_W_ in less than 1 h.

## Materials and methods

### Subjects

Four hundred and twenty six exercise tests (360 subjects, aged 14–69 years (31 ± 10 years), 36 women), conducted between 1997 and 2017, were selected for LT and MLSS_W_ determination. For the field test in running, 47 subjects (2 women) between 20 and 56 years (36 ± 9 years) were recruited. Each participant gave written, informed consent after being provided a detailed description of the study requirements and procedures. The experimental procedures were approved by the University of Konstanz Institutional Review Board and were performed in accordance with the ethical standards of the Government of Baden Württemberg. All subjects were healthy and were instructed to avoid heavy exercise and to maintain a normal diet at least 2 days before testing.

### Study protocol of laboratory tests

**Figure 1:**
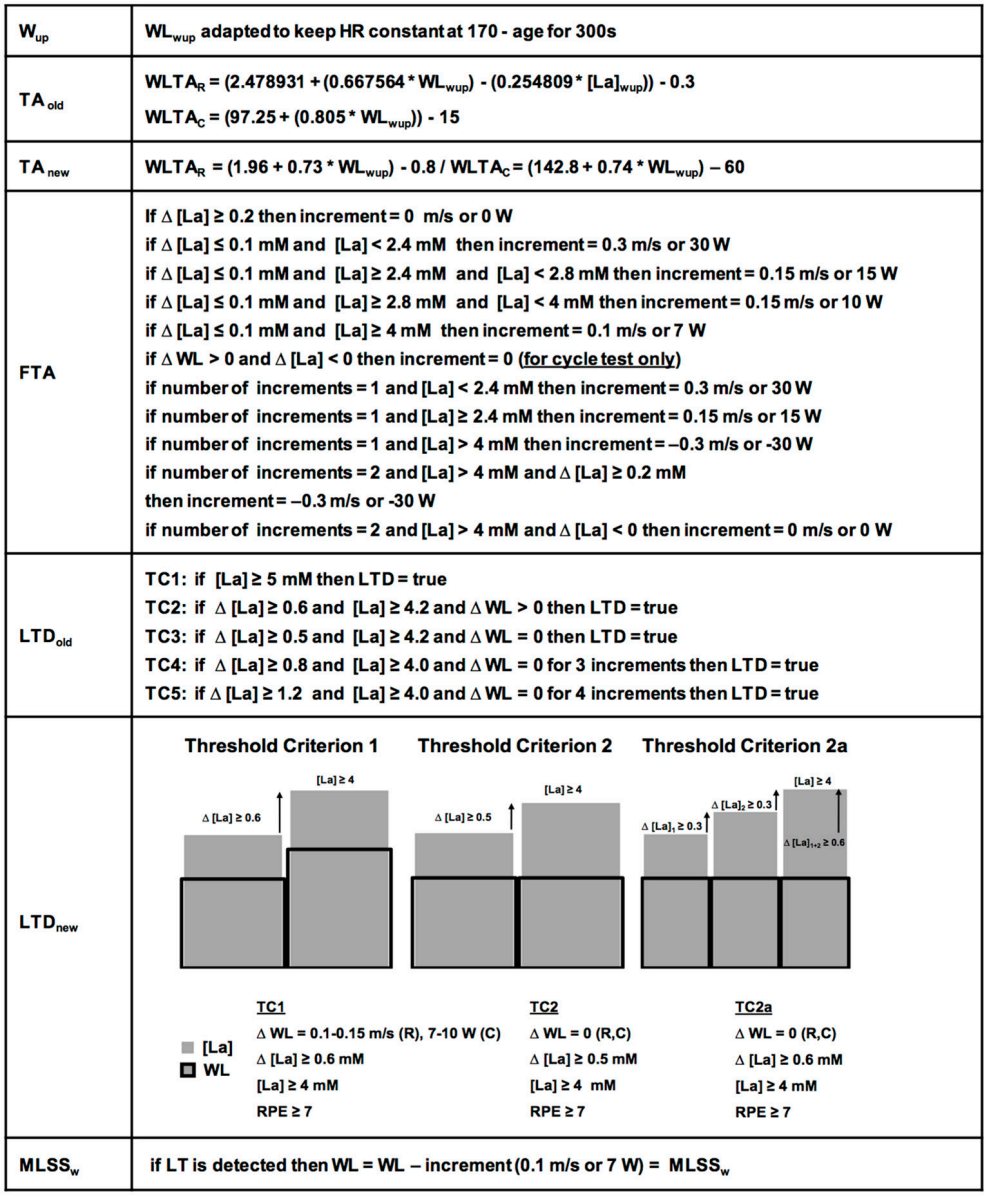
Detailed ILT-Test algorithms. The warm-up (W_up_) workload (WL) was kept constant automatically. The initially used threshold adaptation (TA_old_) algorithm (for determination of the first workload increment after the W_up_), has been continuously developed into the currently used threshold adaptation algorithm (TA_new_). The fine threshold adjustment (FTA) was depending on the number of steps and the lactate concentration after every step. Note the specific criteria for ILT-Tests on the bicycle ergometer for the FTA. The first identified threshold criteria (TCs) for determination of the lactate threshold (LTD_old_), were constantly verified and developed into the actual TCs 1, 2, and 2a (LTD_new_). After lactate threshold (LT) detection, the arterial lactate concentration [LA] increase stopped after a slight workload reduction. HR, heart rate; MLSS_W_, maximal lactate steady state workload; RPE, rating of perceived exertion; WLTA_R_, workload threshold adaptation running; WLTA_c_, workload threshold adaptation cycling.

**Figure 2:**
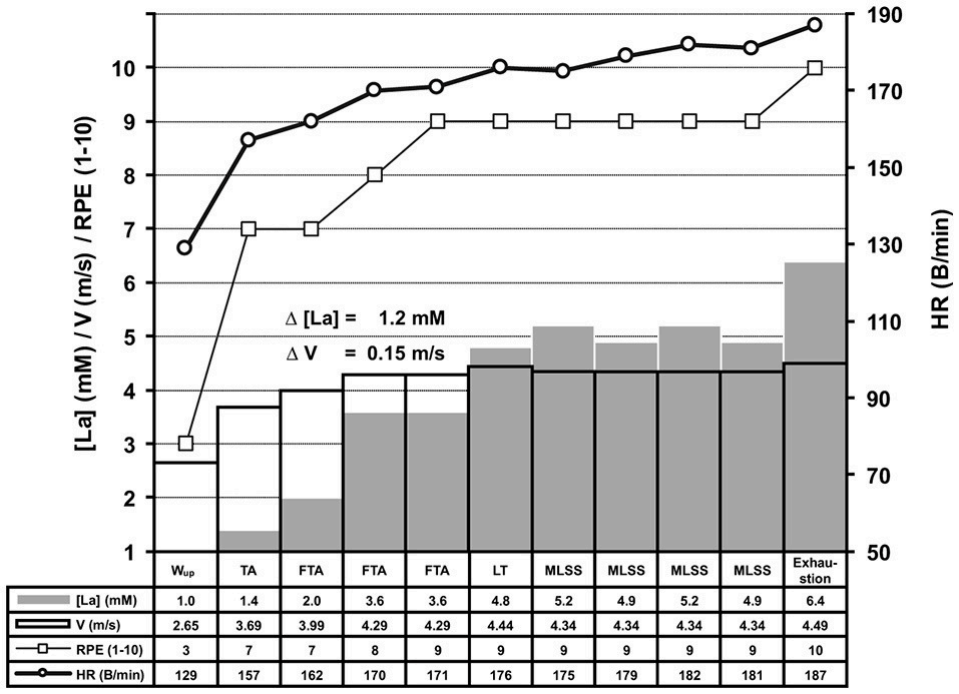
Experimental protocol of the ILT-Test. The basic experimental protocol was identical on the treadmill and bicycle ergometer (for detailed information see Figure 1). After the warm-up (W_up_), threshold adjustment (TA), and fine threshold adjustments (FTA), a steep arterial lactate accumulation occurred at the lactate threshold (LT) (Δ [La] = 1.2 mM), in response to a slight increase of treadmill speed. The arterial lactate accumulation stopped after a small workload (WL) reduction (0.1 m/s), representing the MLSS_W_, and rapidly rose again after another WL increment (exhaustion). Because each WL adjustment based on the lactate level from the previous 4 min step, the duration of the test can vary intraindividually (data not shown). HR, heart rate; [La], arterial lactate concentration; RPE, rating of perceived exertion; V, treadmill velocity.

The basic test protocol was identical on the treadmill (*n* = 278) and bicycle ergometer (*n* = 148) (for detailed explanation see below and Figures 1, 2) and did not change during the data acquisition period. A specific algorithm was developed for every phase of the test (Figure 1). Determination of the LT was carried out, by using so-called “threshold criteria” (TCs) (Figure 1). To improve their clarity and stringency and to ensure a precise LT adaptation during the test (TA, Figures 1, 2), the initial algorithms (TA_old_, LTD_old_) were continuously modified and developed into the current ones (Figure 1, TA_new_, LTD_new_). For the present study, all 426 datasets were retrospectively analyzed with the new TCs. However, it is important to note, that the results were the same, irrespective of the algorithm used.

Each subject performed a warm-up (W_up_) on a custom-built treadmill (Woodway GmbH, Weil am Rhein, Germany; 0% incline), or a modified bicycle ergometer (Lode Excalibur, Lode B. V., Groningen, The Netherlands; equipped with a Powertec bipedal force measurement device, O-tec, Bensheim, Germany; with custom made optical rotatory distance registration) at a heart rate (HR) of 170-age which was kept constant by automated HR-related adjustment of treadmill belt speed or pedal resistance (Figure 1, W_up_).

To assess lactate kinetics at the MLSS_W_, the exercise intensity should rapidly reach the appropriate WL range (WL, treadmill velocity/pedal resistance). Therefore, we estimated the size of the first increment with a regression equation obtained from pilot experiments (Figure 1, TA_old_). By incorporating new data this procedure was continuously adapted to optimize the accuracy and to avoid a running speed or pedal resistance far above/below the LT at the beginning of the test (Figure 1, TA_new_). The transition from the W_up_ WL to the running speed or pedal resistance of the first increment was executed in a smooth, ramp like manner. [La] was measured (Arkray Lactate Pro® 1,710, sampling size 5 μl, measuring time 60 s, coefficient of variability (CV) = 3% (Pyne et al., 2000; Baldari et al., 2009; Tanner et al., 2010; Mamen, 2014), after stopping the treadmill for approximately 15 s to collect arterial blood samples from the hyperaemirized earlobe (Finalgon®, Sanovi-Aventis Germany GmbH, Frankfurt am Main, Germany) (Dassonville et al., 1998; Feliu et al., 1999). Afterwards, the test was continued at the pre-rest velocity until the blood sample was analyzed.

The fine-tuning towards the LT was achieved by means of several steps with slight WL increments until a TC was reached (Figures 1, 2). Every WL increment/reduction was set in accordance to the lactate concentration from the previous step (Figure 1). We favored a 4 min step duration, to ensure a metabolic and blood circulation adaption phase of at least 3 min after a potential WL variation (Weltman et al., 1990; Bentley et al., 2007).

Figure 1 depicts the algorithms for the threshold adaptation (TA), the fine threshold adaptation (FTA) and the lactate threshold detection (LTD). After detection of the LT the running velocity or pedal resistance was slightly reduced (0.1 m/s/7 W) to stop the lactate increase and to verify the MLSS_W._ The HR was recorded simultaneously and a rating of perceived exertion (RPE) was given by the subjects at the end of each increment. The participants were advised to report the exercise level that could be maintained for 60 min as 5 out of 10 in the modified Borg scale (Borg, 1982).

To increase the accuracy of the “individual lactate threshold test” (ILT-Test), to shorten the test duration and to simplify data analysis, we continuously refined the test algorithms (Figure 1). Finally, we found two algorithms with the respective TCs, which could be used to reliably detect the steep [LA] increase at the LT, and thus, allowed a reliable determination of the MLSS_W_ (Figure 1).

**Figure 3:**
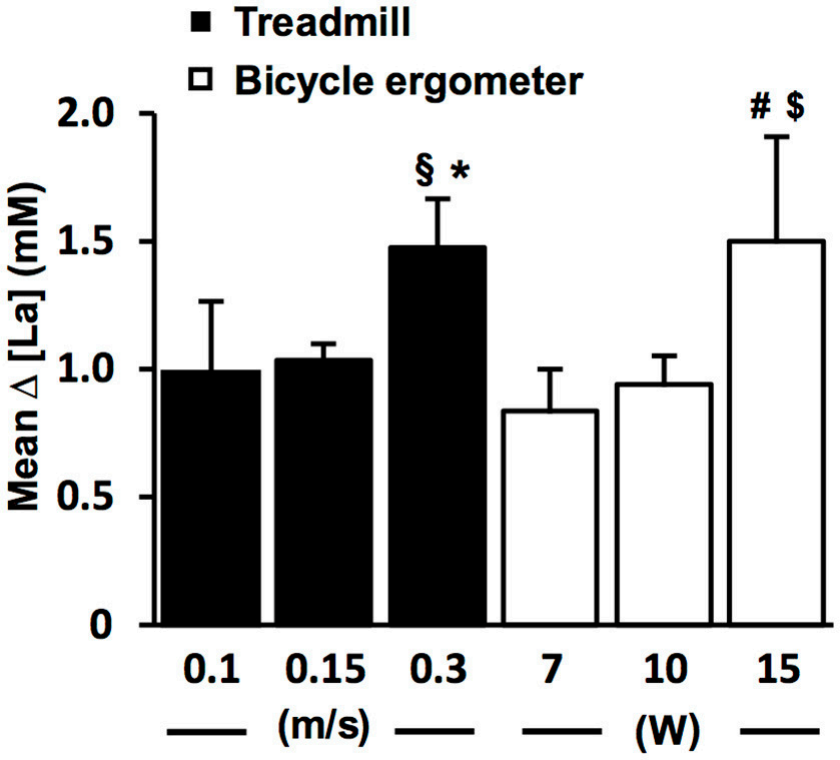
The lactate increase is augmented with higher increments at the individual lactate threshold. Relationship between workload (WL) increment (ΔV, ΔP) and arterial lactate increase (Δ [La]) at the lactate threshold (LT) detected with threshold criterion 1 (TC1) on the treadmill (*n* = 169, black bars) and bicycle ergometer (*n* = 69, white bars). The [LA] increase starts to appear after a slight workload increment of 0.1–0.15 m/s in running and 7–10 W in cycling, and gets more pronounced with higher workload increments. ^§^*P* = 0.02 vs. 0.15 m/s (two-tailed unpaired *t*-test); *P < 0.001 vs. 0.15 m/s (Mann-Whitney-U-Test); ^#^*P* = 0.002 vs. 7 W (Mann-Whitney-U-Test); ^$^*P* < 0.001 vs. 10 W (two-tailed unpaired *t*-test). Data are expressed as means ± SE.

To study the relationship between the size of the [La] increase and the different WL increments at the LT (Figure 3), we performed a more detailed analysis on TC1 datasets (Figure 1). The different WL increments are the result of the fine threshold adaption (FTA) algorithm (Figure 1). To reduce the test duration, it is necessary to rapidly reach the LT. Therefore, the FTA algorithm is designed to reduce the WL increments successively, when the [La] starts to increase in the region of the assumed LT (Figure 1). Datasets with 0.3 m/s and 0.1 m/s WL increments at the LT are the result of pilot experiments on the treadmill with different FTA algorithms (Figure 3).

**Figure 4:**
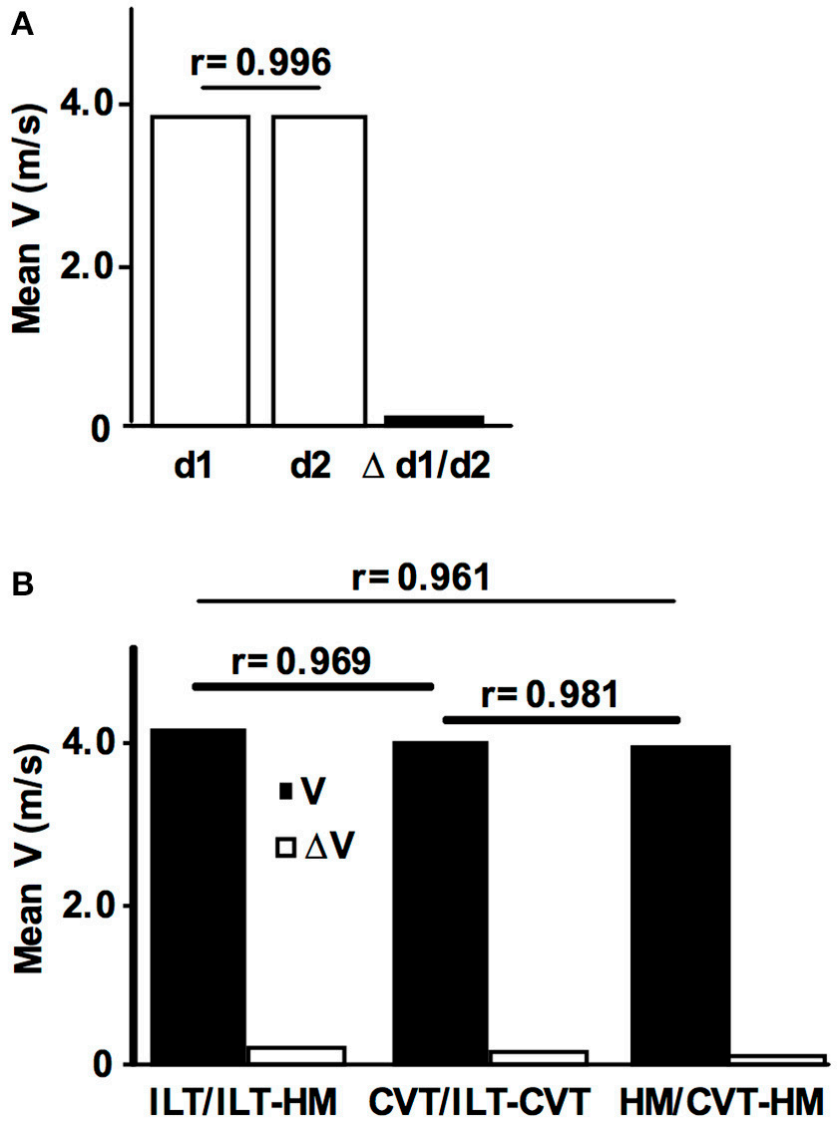
Reliability and validity of the ILT-Test. **(A)** Intraindividual day-to-day comparison of the maximal lactate steady state workload (MLSS_W_) obtained from two ILT-Tests (*n* = 17, MLSS_w_ d1 = 3,77 m/s, MLSS_w_ d2 = 3,76 m/s, Δ d1/d2 = 0.07 m/s). **(B)** Comparison of the mean running speed at the MLSS_W_ obtained from (i) individual lactate threshold tests (ILT-Test), (ii) constant velocity tests (CVT), and (iii) half marathon field tests (HM) (*n* = 47, mean velocity ILT, 4.07 m/s; CVT, 3.93 m/s; HM, 3.86 m/s). Note the only slight differences between tests (ILT/HM, 0.21 m/s; ILT/CVT, 0.14 m/s; CVT/HM, 0.07 m/s). Data are expressed as means. V, treadmill velocity.

The day-to-day reliability for treadmill tests was assessed by repeating the ILT-Test within a time window of 2 days to 2 weeks (*n* = 17) (Figure 4A). Validity was assessed in a 10 km treadmill constant velocity test (CVT) (*n* = 47) (Figure 4B). Here, the initial treadmill speed was set 0.2 m/s below the previously assessed velocity at the LT and increased by 0.1 m/s when the [LA] was constant over two consecutive 2 km steps.

The test-protocol, data analyses and documentation were executed automatically by custom written software. The digitized WL and HR data were recorded at 2 KHz, averaged across 5 s intervals and stored together with lactate levels, RPE, anthropometric and time dependent data.

### Field test study protocol

Before the field test (HM, half marathon race, 21,0975 km, *n* = 47), all subjects conducted an ILT-Test and a CVT as described above. No instructions and no information about the laboratory test results were given to the subjects before the race. The finish time was compared to the ILT-Test and CVT results. The running course was flat, officially measured and paved completely (DJK, 2017).

### Statistical analysis

Statistical analyses were run in SPSS 24.0 (IBM). Significance of differences between distinct WL increments at the LT was analyzed using two-tailed *t*-tests. If the assumptions of normal distribution and homogeneity of variance were violated, the Mann-Whitney-*U*-Test was used. Associations between variables were assessed using Pearson's correlation coefficient. Data are shown as means ± SD unless otherwise stated, and *P*-values of < 0.05 were considered statistically significant.

## Results

**Figure 5:**
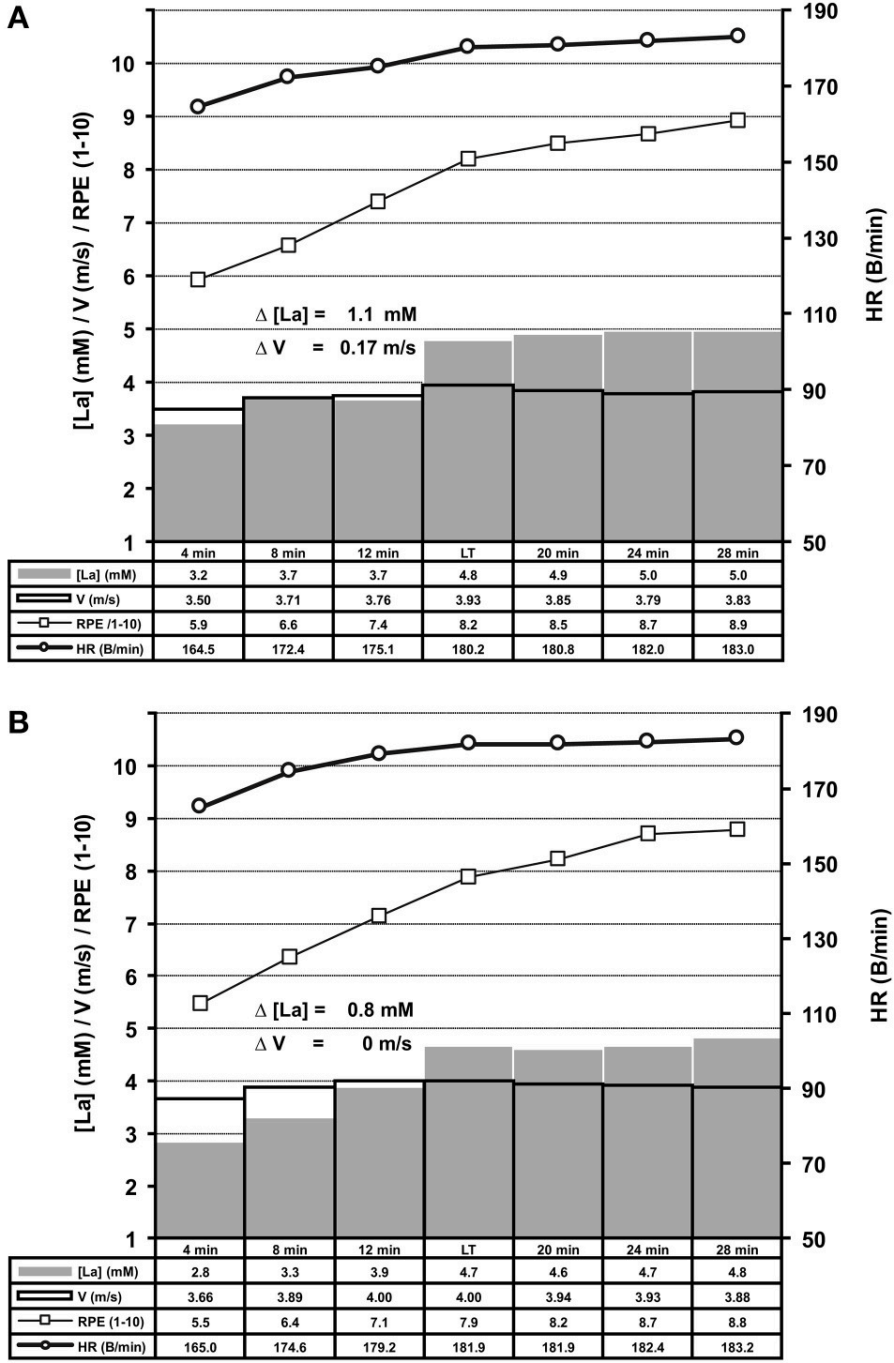
Summary of treadmill ILT-Tests which met threshold criterion 1 (**A**, *n* = 169) or 2 (**B**, *n* = 109). Group mean values are shown for arterial lactate concentration (mM) [La], velocity (m/s), heart rate (B/min) and RPE (1–10). Note the steep [La] increase at the lactate threshold (LT) in response to a slight increase of treadmill velocity. **(A)** Threshold criterion 1, Δ [La] = 1.1 ± 0.44 mM, Δ V = 0.17 ± 0.06 m/s; (**B)** Threshold criterion 2, Δ [La] = 0.8 ± 0.36 mM. HR, heart rate; RPE, rating of perceived exertion; V, treadmill velocity.

**Figure 6:**
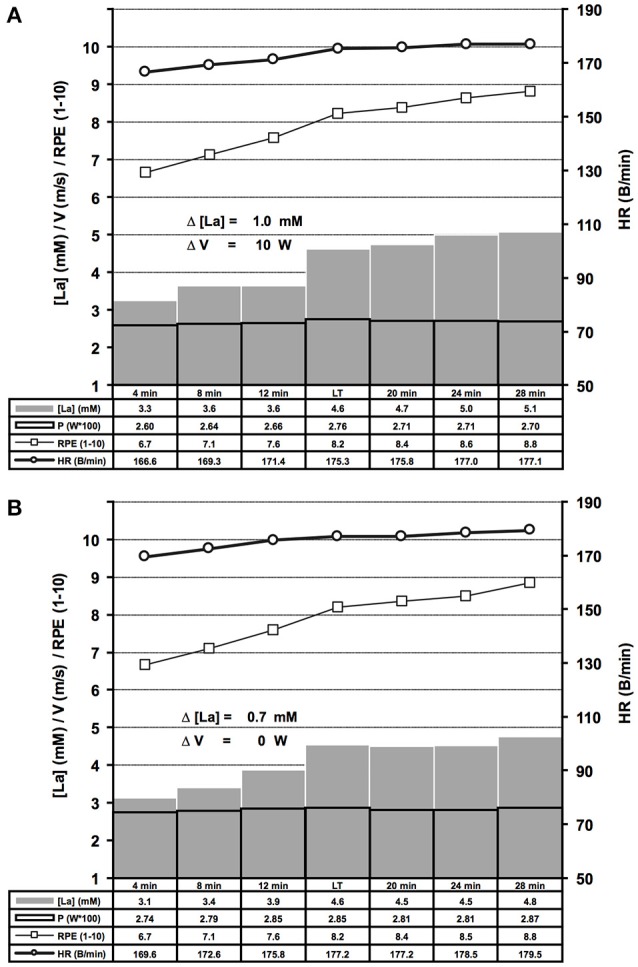
Summary of bicycle ergometer ILT-Tests which met threshold criterion 1 (**A**, *n* = 69) or 2 (**B**, *n* = 79). Group mean values are shown for arterial lactate concentration [La] (mM), velocity (m/s), heart rate (B/min) and RPE (1–10). Note the steep [La] increase at the lactate threshold (LT) in response to a slight increase of cycling power. **(A)** Threshold criterion 1, Δ [La] = 1.0 ± 0.48 mM, Δ W = 10 ± 1.58 W; **(B)** Threshold criterion 2, Δ [La] = 0.7 ± 0.29 mM. V, treadmill velocity.

**Figure 7:**
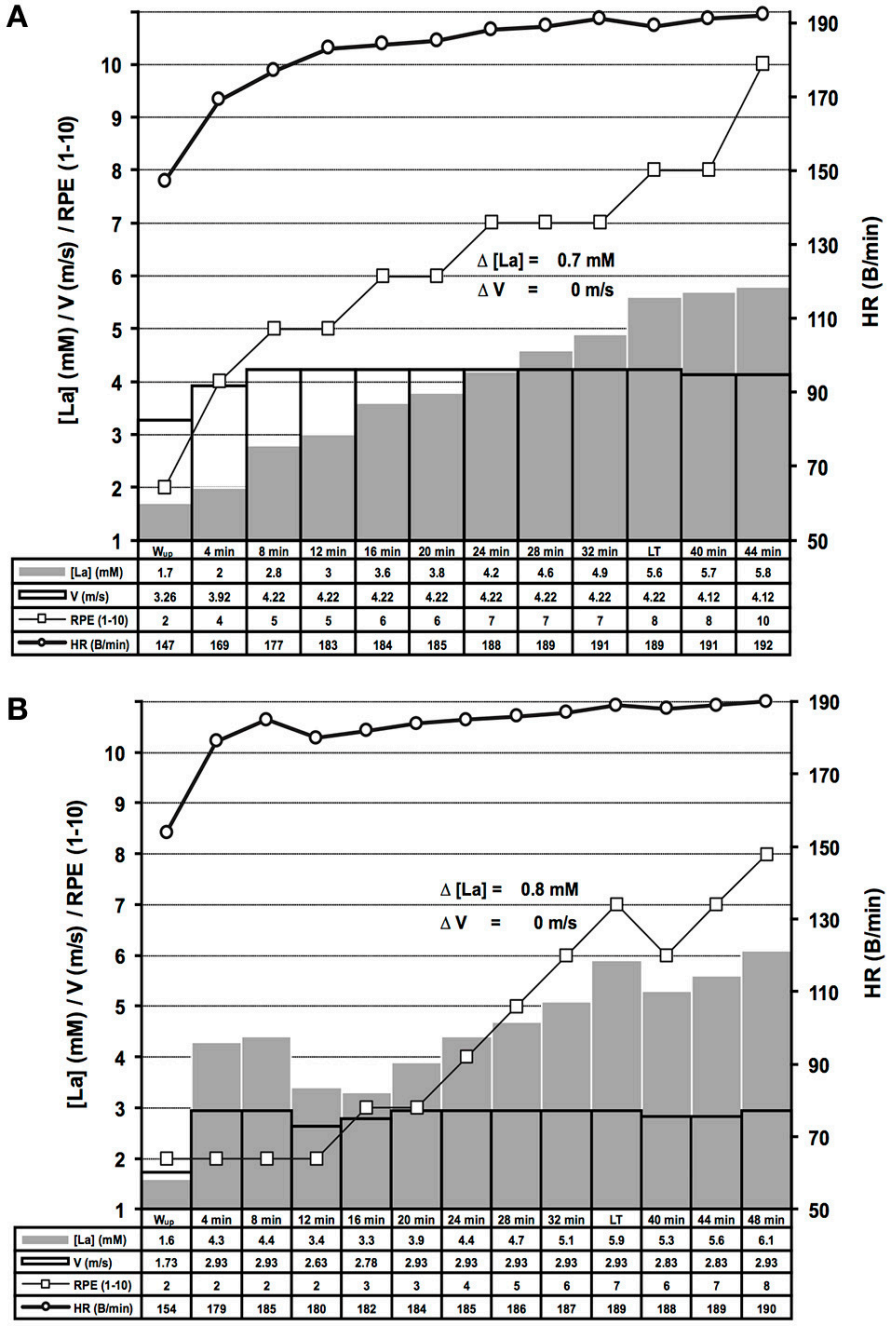
Examples of distinct lactate kinetics near the maximal lactate steady state workload. **(A)** The lactate threshold (LT) appeared after 8 steps of unaltered workload (WL) (threshold criterion 2 (TC2), for rationale see Figure 1); **(B)** After a too high WL at the beginning of the test and subsequent correction, the LT was detected using TC2. HR, heart rate; [La], arterial lactate concentration; RPE, rating of perceived exertion; V, treadmill velocity.

We determined the LT in 278 treadmill and 148 bicycle ergometer single exercise tests, by means of slight WL variations at the MLSS_W_ (Figures 2, 5A,B, 6A,B, 7A,B). The ILT-Test consists of distinct stages: (i) warm-up (W_up_), (ii) threshold adaptation (TA), (iii) fine threshold adaptation (FTA), (iv) lactate threshold detection (LTD) by means of two threshold criteria (TCs), followed by (v) a minimal workload reduction for verification of the MLSS_W_ (Figures 1, 2, 5A,B, 6A,B, 7A,B) (see “Methods” section).

Repeated adaptation of the TC for treadmill and bicycle ergometer tests revealed two scenarios of lactate accumulation at the LT. Either the [LA] increased more than 0.5 mM in the step directly after a WL increment (TC1), or the [LA] increased more than 0.4 mM one to eight steps after the initial WL increment (TC2) (Figure 1). Both, TC1 and TC2 were characterized by a steep increase of [LA] at the LT, which occurred between absolute lactate levels of 1.9 mM and 5 mM, with a mean increase of (0.9 ± 0.43 mM) and a maximum rise of 2.4 mM (Figures 5A,B, 6A,B). Only in 10 out of 426 tests, the [LA] increase did not reach 0.5 mM, but increased slowly within a minimum of 3 consecutive steps of constant workload (TC2a) (Figure 1).

Interestingly, the steep [LA] increase at the LT occurred at different absolute lactate levels in subjects who completed more than one ILT-Test (data not shown).

After LT detection, a small workload reduction (0.1 m/s/7 W) usually stopped the lactate accumulation and was used for MLSS_W_ verification (Figures 1, 2, 5A,B, 6A,B, 7A,B). The RPE increased or kept constant at a high subjective fatigue level after reaching the LT, whereas the HR increased only slightly (Figures 2, 5A,B, 6A,B, 7A,B).

The sharp [LA] increase at the LT already occurred after workload increments of 0.1 m/s on the treadmill and 7 W on the bicycle ergometer (Figure 3). The lactate increase was augmented with higher increments (0.1 vs. 0.30 m/s, *P* = 0.02; 0.15 vs. 0.30 m/s, *P* < 0.001; 10 vs. 15 W, *P* < 0.001; 7 vs. 15 W, *P* = 0.002; Figure 3). In some rare cases, the WL after the TA was already above the MLSS_W_ (Figure 7B). However, after a WL correction at the beginning of the test, it was still possible to detect the LT using TC1 or TC2 and to subsequently verify the MLSS_W_ (Figure 7B).

To examine the reproducibility of the ILT-Test, 17 subjects repeated the protocol after an adequate period of recovery (Figure 4A). The mean day-to-day difference of the running velocity at the MLSS_W_ during the ILT-Test was 0.07 m/s, and a significant positive correlation was found between treadmill velocities at test day 1 and 2 (*r* = 0.996, *P* < 0.001, Figure 4A). Therefore, the mean day-to-day difference was below the workload increments of 0.1–0.15 m/s used in the ILT-Test on the treadmill, suggesting a high reproducibility of the MLSS_W_.

To assess the validity of the ILT-Test in running, we verified the results in a 10 km CVT and in a field test (half marathon race). 47 subjects performed all three tests with sufficient time for regeneration between the exercise loads. We found minimal velocity differences and consequently significant positive correlations between mean velocities from the three test settings (ILT vs. HM, Δ V = 0.21 m/s, *r* = 0.961, *P* < 0.001; ILT vs. CVT, Δ V = 0.14 m/s, *r* = 0.981, *P* < 0.001; CVT vs. HM, Δ V = 0.07 m/s, *r* = 0.969, *P* < 0.001; Figure 4B).

## Discussion

We examined the consequences of slight workload variations at the MLSS_W_ on lactate kinetics in 426 treadmill and bicycle ergometer tests. Within this WL range, increments of 0.1–0.15 m/s on the treadmill or 7–10 W on the bicycle ergometer caused a steep increase of [La] between 0.5 and 2.4 mM indicating LT achievement. The rapid increase of [La] occurred from one to eight 4 min-increments after the initial workload change. In addition to proving that the LT and the MLSS_W_ occur in a narrow WL range, we showed that peripheral arterial blood collection is sufficiently precise for assessing lactate kinetics in response to repeated, minimal WL modifications.

The rapid lactate accumulation at the LT can be explained by a reduced aerobic energy supply as a consequence of insufficient O_2_ delivery (Wasserman et al., 1973; Heck et al., 1985; Mader and Heck, 1986). However, considerable evidence indicates that this phenomenon is a consequence of a limited ability of body tissues to use it as a source of oxidative energy production and/or gluconeogenetically for the restoration of glycogen stores (Brooks, 1985a,b; Stainsby and Brooks, 1990). Starting with the work by Stainsby and Welch (1966), and followed by other animal (Connett et al., 1984, 1986; Brooks, 1985b; Stainsby and Brooks, 1990) and human studies (Donovan and Brooks, 1983; Richardson et al., 1998; Messonnier et al., 2013), evidence suggests that blood lactate appearance is not the effect of a O_2_ lack *per se* (Stainsby and Brooks, 1990). Rather, the discovery of intracellular- and cell-to-cell shuttle systems (Brooks, 2009) provides a framework for the basic understanding of lactate kinetics at the LT and the MLSS_W_ (for comprehensive reviews see (Stainsby and Brooks, 1990; Gladden, 1996, 2000, 2004, 2008). Cell-to-cell shuttles mediate intramuscular lactate exchange between diverse fiber types, between different muscle subtypes (heart/skeletal), and between tissues of net lactate release and gluconeogenesis (lactate consuming tissues; Emhoff et al., 2013). The transport between sites of lactate production and consumption is largely mediated by two isoforms of mono carboxylate transport proteins (MCT). MCT1 is supposed to mediate lactate uptake into the cell, whereas MCT4 is capable of removing lactate from the cell (Brooks et al., 1999; Dubouchaud et al., 2000; Kobayashi, 2004; Hashimoto and Brooks, 2008). In this context, intra- and interindividual differences in fiber type distribution of leg muscles involved in running and cycling have to be considered (Johnson et al., 1973; Hering, 2000). The functionally diverse motor units are normally classified into three broad categories (Type I, IIa, IId/x) according to their physical, metabolic or myosin heavy chain profile (Brooke and Kaiser, 1970; Pette and Staron, 1997, 2001). Additionally, Henneman and colleagues showed that all fiber types are reflex-controlled on the spine level with a fixed recruitment order (Henneman and Olson, 1965; Henneman et al., 1965). This so called “size principle” implies that Type I fibers (small size, low force production, aerobic energy metabolism, fatigue resistant), become initially recruited during force development. When higher force production becomes necessary, IIa fibers (larger size, medium force production, more glycolytic energy metabolism, fatigue resistant) become recruited, followed by 2d/x motor units (largest size, high force production, glycolytic energy metabolism), when maximal force production becomes necessary. 2d/x motor units are usually not recruited during long lasting and repetitive twitches (Vøllestad and Blom, 1985). In contrast to other fiber types they are characterized by a reduced amount and smaller mitochondria (Ingjer, 1979), sparse MCT1 (Kobayashi, 2004), low levels of aerobic enzymes (Peter et al., 1972) and a low density of surrounding capillaries (Ingjer, 1979). In contrast, they possess a higher glycolytic capacity (Peter et al., 1972) and have a higher activity of MCT4 (Kobayashi, 2004). Importantly, evidence from animal (Armstrong et al., 1974; Dudley et al., 1982) and human studies (Vøllestad et al., 1984; Vøllestad and Blom, 1985; Green et al., 1990; Nordheim and Vøllestad, 1990) supports the assumption of an increased type IIa and Type IId/x fiber recruitment, if the WL is in the range of the MLSS_W_. Additionally, human tracer experiments showed, that the metabolic clearance rate (MCR) for lactate rises at the transition from rest to moderate intensity exercise, but fells when workload demands get close to the LT, pointing to a limited capacity of endogenous lactate clearance systems (Messonnier et al., 2013).

What happens if the muscle power for a given running velocity or pedal resistance is mainly provided by Type I fibers with only minor contribution of Type II fibers? Previous data suggests that blood lactate levels will be constant at low levels because via cell-to cell-shuttle systems or the influx from capillaries, the lactate of the more glycolytic IIa fibers or other tissues will be oxidized within type I fibers. What happens if the portion of IIa fibers for a given workload increases? The blood lactate concentration will be constant too, but at higher levels because the MCT4 mediated efflux from type II fibers into the blood stream increases (Brooks et al., 1999; Dubouchaud et al., 2000; Kobayashi, 2004; Hashimoto and Brooks, 2008). Under a WL, at which force demands of the recruited muscle cells only slightly exceed the energy costs for the already active motor units, it is likely that the nervous system enhances the innervation frequency and/or activates additional muscle fibers (Vøllestad et al., 1984; Vøllestad and Blom, 1985).

In the case of saturated lactate elimination and a constant WL, the latter two scenarios will cause a slowly increasing lactate concentration until fibers in one or different muscles have to be supplemented by larger, high glycolytic ones.

In elite cyclists, Lucía et al. (1999) found two electromyographic thresholds (EMG_T_) for the vastus lateralis and rectus femoris. The EMG_T1_ appeared around 66% of the maximal oxygen uptake (VO_2max_) and might reflect the increasing input of type IIa fibers, followed by a second notable increase in EMG activity around 87% VO_2max_ (EMG_T2_), which might reflect the recruitment of the largest and probably not aerobically trained motor units. The latter ones are likely also responsible for the rise in [La] at the LT.

Additionally, the epinephrine and norepinephrine status and resulting blood flow are important co-factors influencing the lactate kinetics (Stainsby et al., 1985; Martin et al., 1989a,b; Stainsby and Brooks, 1990). Together, these considerations provide a framework for our findings of a fine-tuned lactate metabolism at the MLSS_W_, the variable test duration even in intraindividual comparison, and the variable LT appearance from one up to 8 steps after the initial workload increment.

Lactate acidosis was a long-held model for the fatigue at WLs above the MLSS_W_ (Fitts, 1994). But experiments with McArdle disease patients in the 1990s provided substantial evidence that fatigue can occur without lactate production (Cooper et al., 1989) and that a lactate induced acidosis does not largely affect force production in skinned muscle fibers (Chase and Kushmerick, 1988). In contrast, it has been demonstrated that lactate can have a fatigue reducing effect (Allen and Westerblad, 2004; Pedersen et al., 2004). Consequently, factors other than lactate were identified that are capable of eliciting a reversible force loss, defined as fatigue (Allen et al., 2008). This includes metabolic processes around the excitation-contraction coupling process (Westerblad et al., 2002; Katz and Westerblad, 2014), and a diminished phosphorylation potential (Pette and Vrbová, 2017). If the level of force production induces the recruitment of large glycolytic fiber types, the endurance time is very likely limited due to the high energy demands and low aerobic capacity of these fibers (Brooke and Kaiser, 1970; Peter et al., 1972). Furthermore, these factors impair the function of the calcium pumps and extend the intracellular retention period of calcium, which is discussed as an important factor for muscle fiber plasticity, in particular a MHC protein transformation from fast to slow (simplified scheme type 2d/x → type IIa → type I) (Pette and Staron, 1997, 2001; Pette, 1998; Pette and Vrbová, 2017). The slower frequency of cross-bridge cycles of the transformed myosin is also discussed as a way to save energy (He et al., 2000). This pattern of plasticity is generally accepted for animal muscle stimulation experiments and for 2d/x to IIa transformation in humans (Saltin et al., 1995a). However, only limited data supports the transformation from type IIa to type I *in vivo* (Jansson et al., 1978; Howald et al., 1985; Dubouchaud et al., 2000). Additionally, the amount of MCT1 in Type I and Type IIa fibers and of MCT1 and MCT4 in Type IId/x increase as a consequence of training at MLSS_W_ (Dubouchaud et al., 2000; de Araujo et al., 2015), accompanied by an enhanced metabolic clearance rate (MCR) (Bergman et al., 1999; Messonnier et al., 2013). We found a negative correlation between the [La] at test minute 4 (*r* = −0.30; *P* < 0.001, data not shown), 8 *(r* = −0.26, *P* < 0.001, data not shown), 12 (*r* = −0.19, *P* < 0.01, data not shown), the LT (*r* = −0.17, *P* < 0.01, data not shown), and the velocity at MLSS_W_ in all treadmill tests. Together with significant lower [La] of elite Kenyan runners compared to elite Scandinavian runners (Saltin et al., 1995b), this points to a relationship between reduced lactate levels below the MLSS_W_, muscle fiber type distribution, movement economy and the endurance capacity in running and cycling.

In a study with world class riders, cycling economy (CE) [W * (LO_2_/min)^−1^] was found to be an important factor to explain the extraordinary endurance capabilities of champions (Lucía et al., 2002). Lucia and colleagues found an inverse relationship between the VO_2__max_ in a ramp protocol and CE at 80% VO_2_ max in a constant WL test. Furthermore, Coyle and co-workers revealed a significant positive relationship between the amount of type I fibers and CE (Coyle et al., 1992), and Krustrup et al. found an elevated VO_2_ during submaximal work in humans, when type I fibers were blocked with cis-Atracurium (Krustrup et al., 2008).

Therefore, it is tempting to speculate that the adequate training stimulus to improve the fatigue resistance of the “non-endurance trained” type II fibers, has to be in the region of MLSS_W_ with progressive duration, which is supported by data from animal and human training studies (Dudley et al., 1982; Bergman et al., 1999; Billat et al., 2004; Messonnier et al., 2013).

### Summary

We show that the precise detection of the LT is a prerequisite for MLSS_W_ determination. The steep arterial lactate accumulation at the LT was stopped by decreasing the WL by 0.1 m/s or 7 W respectively, resulting in a constant [La] under reduced physical effort. This post-threshold level considered as MLSS or MLSS_W_ was validated in a series of 10 km treadmill runs at constant velocity (CVT), in which [LA] was controlled to achieve a steady state (r_ILT/CVT_ = 0.981) and in a half marathon race (r_CVT/HM_ = 0.969).

Although the number of increments and the absolute [LA] during the ILT-Test can vary on a day-to-day basis, even intraindividually, the lactate threshold (LT) is highly reproducible (r _test/re−test_ = 0.996). A common explanation for the increasing [La] at the LT is the imbalance between production and removal within the complex shuttle network of lactate metabolism (Donovan and Brooks, 1983; Brooks, 1985a; Stainsby and Brooks, 1990; Messonnier et al., 2013). During the ILT-Test, the arterial lactate accumulation did not occur in a curvilinear manner as described for graded test protocols (Bentley et al., 2007). The lactate enhancement occurs at a specific WL with no relation to the absolute [La], nor to the number of previous steps. These data suggest that enhanced activity in motor units (e.g., close to the MLSS_W_) within muscles with high lactate efflux caused an accelerated arterial lactate accumulation in relation to power output, when activity in lactate consuming motor units was unchanged. The change in neuronal activity at exercise intensities near MLSS_W_ can occur intramuscularly (Armstrong et al., 1977; Armstrong and Taylor, 1982; Armstrong and Laughlin, 1985; Lucía et al., 1999) or might be mediated between muscles with different fiber distributions, as described indirectly by mitochondrial adaptations to different training intensities in rats (Dudley et al., 1982), and may also contribute to the rapid arterial lactate accumulation at the LT.

## Conclusions

We used minor arterial lactate concentration-dependent workload (WL) modulations at the maximal lactate steady state workload (MLSS_W_) on the treadmill and bicycle ergometer to precisely detect the lactate threshold (LT). LT achievement was characterized by a steep arterial lactate accumulation after a minor WL increase. A subsequent WL reduction resulted in the stabilization of lactate levels and was used for MLSS_W_ determination. We found a high reproducibility of the running velocity at the MLSS_W_ on a day-to-day basis and a high correlation between running velocities during ILT-Tests, 10 km constant velocity tests and a half marathon race field test. Thus, the ILT-Test allows a reliable and valid determination of the MLSS_W_. The remarkable sensitivity of the lactate metabolism in response to minor WL changes, in combination with previous data argues for adaptations in muscular innervation patterns at the MLSS_W_. The cellular mechanisms of lactate transportation and metabolism are located in muscle fibers and may be neuronally tuned in relation to mechanical demands (Armstrong and Laughlin, 1985). We conclude that arterial lactate kinetics might reflect force control and fatigue, without being causally involved.

## Author contributions

GH developed the hard- and software setup, conceived and designed research, performed experiments, analyzed data, prepared and interpreted results, drafted manuscript, prepared figures, edited and revised manuscript; EH and HR consulted study design, edited and revised manuscript; JS performed experiments, interpreted results, prepared figures, edited and revised manuscript, approved final version of manuscript.

### Conflict of interest statement

The authors declare that the research was conducted in the absence of any commercial or financial relationships that could be construed as a potential conflict of interest.
